# Real-World Clinical Outcomes With Sacituzumab Govitecan in Metastatic Triple-Negative Breast Cancer

**DOI:** 10.1200/OP.24.00242

**Published:** 2024-10-01

**Authors:** Sabah Alaklabi, Arya Mariam Roy, Paola Zagami, Anumita Chakraborty, Nicole Held, Joseph Elijah, Anthony George, Kristopher Attwood, Saba S. Shaikh, Lubna N. Chaudhary, Yara Abdou, Shipra Gandhi

**Affiliations:** ^1^ Department of Oncology, Roswell Park Comprehensive Cancer Center, Buffalo, NY; ^2^ Cancer Center of Excellence, King Faisal Specialist Hospital and Research Center, Riyadh, Saudi Arabia; ^3^ Department of Oncology, University of North Carolina at Chapel Hill, Chapel Hill, NC; ^4^ Department of Medicine, Temple University, Philadelphia, PA; ^5^ Division of Hematology and Oncology, Department of Medicine, Medical College of Wisconsin, Milwaukee, WI; ^6^ Department of Pharmacology, Northeastern University School of Pharmacy and Pharmaceutical Sciences, Boston, PA; ^7^ Department of Biostatistics & Bioinformatics, Roswell Park Comprehensive Cancer Center, Buffalo, NY; ^8^ Department of Medicine, Division of Hematology/Oncology, University of Texas Health San Antonio, San Antonio, TX

## Abstract

**PURPOSE:**

Sacituzumab govitecan (SG) is approved for the treatment of metastatic triple-negative breast cancer (mTNBC). We report the real-world clinical effectiveness and toxicity data of SG in patients with mTNBC.

**METHODS:**

A multi-institution retrospective study of patients with mTNBC treated with SG from January 2021 to May 2023 was conducted. Demographic and clinicopathologic variables were collected. Univariate and multivariate Cox regression models were used for survival analysis.

**RESULTS:**

A total of 115 patients were included. The median age at SG initiation was 60 years (range, 31-85). All patients were female; 73 (63.5%) were White and 31 (27.0%) were Black. The median number of previous therapies in the metastatic setting was two (range, 0-8). Sixty-one (56.0%) patients had primary refractory disease. Median relative dose intensity was 92% (range, 33%-100%). Grade 3 or higher adverse events (AEs) were seen in 50.9% of patients, which included neutropenia (35.7%), anemia (27.0%), vomiting (16.5%), fatigue (8.7%), and diarrhea (7.0%). Dose reductions and treatment discontinuation due to AEs were required in 51.3% and 13.2%, respectively. The objective response rate (ORR) was 27.8%. Median overall survival was 9.6 months (95% CI, 7.8 to 12.9) and median progression-free survival (PFS) was 4.8 months (95% CI, 3.6 to 5.9). In patients with human epidermal growth factor receptor 2 (HER2)–low mTNBC who received trastuzumab deruxtecan (T-DXd) after SG, the ORR to T-DXd was 34.8% and median PFS was 7 months (95% CI, 4.6 to 10.1).

**CONCLUSION:**

In a real-world cohort of heavily pretreated patients with mTNBC, SG retains its clinical activity. In a subgroup with HER2-low disease, T-DXd continues to demonstrate promising clinical activity after SG, supporting the use of sequential antibody-drug conjugates in this population.

## Teaser

Our multi-institutional study demonstrates that sacituzumab govitecan is effective in heavily pretreated patients with metastatic triple-negative breast cancer, maintaining clinical efficacy with manageable toxicity. Additionally, we observed that trastuzumab deruxtecan (T-DXd) shows promising clinical activity in a subgroup with HER2-low disease, even after treatment with sacituzumab govitecan.

## INTRODUCTION

Metastatic triple-negative breast cancer (mTNBC) is associated with poor prognosis because of its aggressive nature with a 5-year overall survival (OS) rate ranging from 4% to 20%.^1-6^ Sacituzumab govitecan (SG), a TROP2-directed antibody-drug conjugate (ADC), was US Food and Drug Administration (FDA)–approved for patients with unresectable locally advanced or mTNBC who had received at least one previous line of therapy, on the basis of significant improvement in OS with SG reported in the ASCENT trial.^7,8^ Randomized clinical trials (RCTs) may not consistently mirror the diverse patient population encountered in real-world clinical settings^9-13^; it is therefore essential to understand the real-world clinical effectiveness of recently approved drugs. Furthermore, sequencing of ADCs in mTNBC with the recent additional approval of trastuzumab deruxtecan (T-DXd), which is an ADC that targets human epidermal growth factor receptor 2 (HER2) and with a topoisomerase I inhibitor (payload similar to SG), has become challenging, given the absence of sequencing data in clinical trials.^14^

**CONTEXT**

**Key Objective**
Does sacituzumab govitecan (SG) hold efficacy in heavily pretreated patients with metastatic triple-negative breast cancer (mTNBC) without excessive toxicity?
**Knowledge Generated**
Our findings indicate that SG maintains its clinical effectiveness in a real-world cohort of heavily pretreated patients with mTNBC. In a subgroup with human epidermal growth factor receptor 2 (HER2)–low disease, trastuzumab deruxtecan (T-DXd) shows continued promising clinical activity after SG, with an objective response rate of 34.8%.
**Relevance**
SG can be used in heavily pretreated patients with mTNBC without significant toxicities. Additionally, T-DXd can be considered for HER2-low TNBC patients who have shown clinical benefit with SG. Our study supports the sequential use of antibody-drug conjugates in selected patients with mTNBC.

We present a retrospective analysis of real-world outcomes of SG in a diverse patient population with mTNBC. Additionally, we report the efficacy outcomes of treatment with T-DXd after progression on SG in a subset of mTNBC patients with HER2-low disease.

## METHODS

This is a retrospective, multi-institutional study that includes patients with mTNBC who were treated with SG between January 2021 and May 2023 at four academic centers in the United States: Roswell Park Comprehensive Cancer Center (RPCCC), Buffalo, NY; University of North Carolina, Chapel Hill, NC; Fox Chase Cancer Center, Philadelphia, PA; and Medical College of Wisconsin, Milwaukee, WI. Adult patients (age 18 years and older) diagnosed with mTNBC and treated with SG were included in the study. Patients with other concomitant cancer diagnosis were excluded. The electronic medical records of SG-treated patients with mTNBC were retrospectively analyzed. Five investigators (S.A., A.M.R., P.Z., N.H., and A.C.) performed manual review of each patient record to obtain the data. Baseline demographic characteristics included age, race, clinical characteristics, Charlson comorbidity index (CCI), Eastern Cooperative Oncology Group (ECOG) performance status, site, and number of organs with metastases, and previous lines of treatment in the metastatic setting. Primary refractory disease was defined as relapse occurring within 12 months after the completion of neoadjuvant or adjuvant therapy for TNBC.^7^ Time from initiation of SG until progression, rate of utilization of primary versus secondary prophylactic granulocyte colony-stimulating factor (GCSF; primary prophylaxis: utilization of GCSF with the first cycle of SG before the onset of neutropenia [within 7 days]; secondary prophylaxis: use of GCSF after first cycle of SG),^15^ adverse events (AEs), and last follow-up were collected. AEs were graded on the basis of the Common Terminology Criteria for Adverse Events version 5.0. In addition, investigators evaluated real-world effectiveness of SG by tumor response assessment according to RECIST v1.1 at intervals as decided by the treating physicians, which were mostly on the basis of clinical judgment and institutional practice (response assessment scans were taken at an interval of 8-12 weeks in our study). The scans were reviewed by radiologists at corresponding institutions for response assessments and crosschecked by treating oncologists. Clinical outcomes of treatment with T-DXd after SG were collected as well, including objective response rate (ORR), progression-free survival (PFS; defined as the time from the start of SG to radiologic or clinical progression), and clinical benefit rate (CBR), which was defined as partial response (PR), complete response (CR), or stable disease (SD) with duration >6 months. The relative dose intensity (RDI) of SG was determined by dividing the total dose administered (in mg/kg) by the expected dose (in mg/kg) multiplied by the number of scheduled doses throughout the patient's treatment duration. Informed consent was not obtained from the included patients as the study was retrospective and no protected health information was collected. The study protocol (BDR 161422) was approved by the independent ethics committees/institutional review boards of all participating centers. The reporting has been done in accordance with the ESMO guidance for reporting oncology real-world evidence.^16^ The analysis was performed by the statisticians at RPCCC (K.A. and A.G).

All statistics were performed using SAS version 9.4 (SAS Institute Inc, Cary, NC). The mean, median, standard deviation, and range were reported for continuous variables, with comparisons made using Mann-Whitney *U* and Kruskal-Wallis tests. For categorical variables, the frequencies and relative frequencies were reported and compared using Fisher's exact tests and Pearson chi-square tests. OS, disease-specific survival (DSS), and PFS were reported using standard Kaplan-Meier methods. Univariate and multivariate Cox regression modeling were used to analyze survival outcomes.

## RESULTS

**FIG 1.**
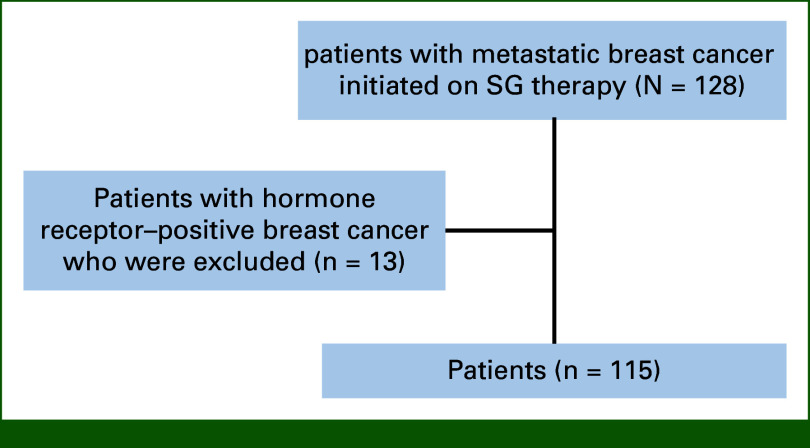
Flow diagram showing patients eligible for the study. SG, sacituzumab govitecan.

A total of 128 patients with metastatic breast cancer who were initiated on SG therapy from January 2021 until May 2023 were identified. Among them, 115 patients met the eligibility criteria, while 13 patients were excluded because of hormone receptor–positive breast cancer (Fig 1). Twenty patients had de novo metastatic breast cancer.

### Demographic, Clinicopathologic, and Treatment Characteristics of the Study Population

The median age at diagnosis with mTNBC was 58 years (range, 29-84), and the median age at the time of start of SG was 60 years (range, 31-85). Of the 115 patients, 76 (66.1%) were age 65 years or younger, while 39 (33.9%) were older than 65 years. All 115 patients in the group were female. Among them, 73 (63.5%) were White, 31 (27.0%) were Black, one (0.9%) was Asian, and 10 (8.7%) were from other nonspecified racial backgrounds. The median CCI score was 8 (range: 6, 12) and the median ECOG score was 1 (range, 0-2).

Primary refractory disease was reported in 61 (56.0%) patients. The median number of previous therapies in the metastatic setting was two (range, 0-8). Ten patients (8.7%) received previous therapy with poly-ADP ribose polymerase inhibitors, and 59 patients (51.3%) received previous therapy with immune checkpoint inhibitors (ICIs). Other previous lines of treatments are given in Table 1.

**TABLE 1.** Baseline Characteristics and Previous Treatments

Variable	Value
Characteristic, No. (%)	115 (100)
Age at start of SG, year, median (range)	60.3 (31.5-85.8)
Age at start of SG, No. (%)	
≤65	76 (66.1)
>65	39 (33.9)
Sex, No. (%)	
Female	115 (100.0)
Race, No. (%)	
White	73 (63.5)
Black	31 (27.0)
Asian	1 (0.9)
Other^a^	10 (8.7)
CCI, median (range)	8.00 (6.00-12.00)
ECOG, median (range)	1.00 (0.00-4.00)
Organs involved with metastasis, No. (%)	
1	16 (14.0)
2	35 (30.7)
3	41 (36.0)
4	16 (14.0)
≥5	6 (5.3)
Metastatic disease location, No. (%)	
Lymph nodes	55 (47.8)
Lung	62 (53.9)
Bone	64 (55.7)
Liver	58 (50.4)
Brain	25 (21.7)
Available biopsy at the time of MBC diagnosis, No. (%)	105 (91.3)
HER2 by IHC, No. (%)	
0	47 (41.6)
1+	43 (38.1)
2+	23 (20.4)
RDI, %, median (range)	92 (33-100)
Starting dose, mg/kg, median (range)	10.00 (5.00-10.00)
Cycles of SG received, No. (%)	
≤3	50 (43.5)
>3	65 (56.5)
Previous lines of therapy in the metastatic setting, No. (%)	
≤3	81 (70.4)
>3	34 (29.6)
Primary refractory disease, No. (%)	61 (56.0)
Previous therapies in the metastatic setting, median (range)	2.00 (0.00-8.00)
Previous PARPi treatment, No. (%)	10 (8.7)
Previous immune checkpoint inhibitor treatment, No. (%)	59 (51.3)
Previous chemotherapy drug, No. (%)	
Taxane	81 (70.43)
Anthracycline	38 (33.04)
Cyclophosphamide	23 (20)
Carboplatin	49 (42.61)
Capecitabine	55 (47.83)
Primary prophylactic GCSF support, No. (%)	39 (33.9)
T-DXd after SG, No. (%)	26 (22.6)
Received local therapy to brain at the time of SG initiation, No. (%)	6 (6.4)

Abbreviations: CCI, Charlson comorbidity index; ECOG, Eastern Cooperative Oncology Group; GCSF, granulocyte colony-stimulating factor; HER2, human epidermal growth factor receptor 2; IHC, immunohistochemistry; MBC, metastatic breast cancer; PARPi, poly-ADP ribose polymerase inhibitor; RDI, relative dose intensity; SG, sacituzumab govitecan; T-DXd, trastuzumab deruxtecan.

^a^ Nonspecified races.

### Histology and Sites of Metastases

Majority (91.3%) of patients underwent a biopsy at the time of metastatic breast cancer diagnosis, which was used for receptor status identification including HER2 status by immunohistochemistry (IHC), and the remainder had receptor status determined from primary tumor pathology. HER2 IHC scores were 0 in 47 patients (41.6%), 43 (38.1%) had HER2 1+, and 23 (20.4%) had HER2 2+ by IHC with nonamplified in situ hybridization. The most common organs involved with metastases were bone (55.7%), lung (53.9%), liver (50.4%), and brain (21.7%). Four patients (16%) received local therapy to the brain at the time of SG initiation, and others had stable brain metastasis at the time of SG initiation.

### Real-World Effectiveness

The ORR with SG treatment was 27.8%. This included 26 (26.8%) patients with PR and one (1.0%) with CR. Additionally, 34 (35%) patients had SD for more than 6 months and 36 (37.1%) had progressive disease. When we analyzed the data on the basis of primary refractory disease, the CBR of those who had primary refractory disease was 35.5% (n = 18) compared with 54.6% (n = 18) in those who did not have primary refractory disease (*P* = .11). Among those who had primary refractory disease, one patient had CR (2%) and seven patients had PR (13.7%). SD was the best response observed in 43% of cases and the median duration of response was 4 months (range, 0-16). Among those who did not have primary refractory disease, 12 patients (36.4%) had PR and 10 patients (30.3%) had SD.

We analyzed the data on the basis of the HER2 receptor status and there was no statistically significant difference in CBR observed across the three HER2 status groups (0, +1, and +2; 48.9%, 35.6%, and 15.6%, respectively; *P* = .4).

Receipt of multiple previous regimens was associated with lower CBR with SG (*P* < .001). The median number of previous regimens for patients with clinical benefit was two (0-7). For patients without clinical benefit, the median number of previous regimens was three (range, 0-8). The median response duration was shorter for patients who had received more than three lines of therapy (2 months) compared with those with three or fewer lines (2 *v* 5 months; *P* = .012).

### RDI of SG

The median RDI of SG was 92% (range, 33%-100%). The median starting dose of SG was 10 mg/kg, with a range of 5-10 mg/kg. Sixty-five patients (56.5%) received more than three cycles of SG at the time of data cutoff, while 50 (43.5%) received three or fewer cycles. The median starting dose for patients who had objective response, SD, and progression of disease (PD) was 10 mg/kg, with a range of 5-10 mg/kg. There was no statistically significant difference between RDI, median starting dose, and ORR or CBR (Data Supplement, Table S1, online only).

### Survival Outcomes

**FIG 2.**
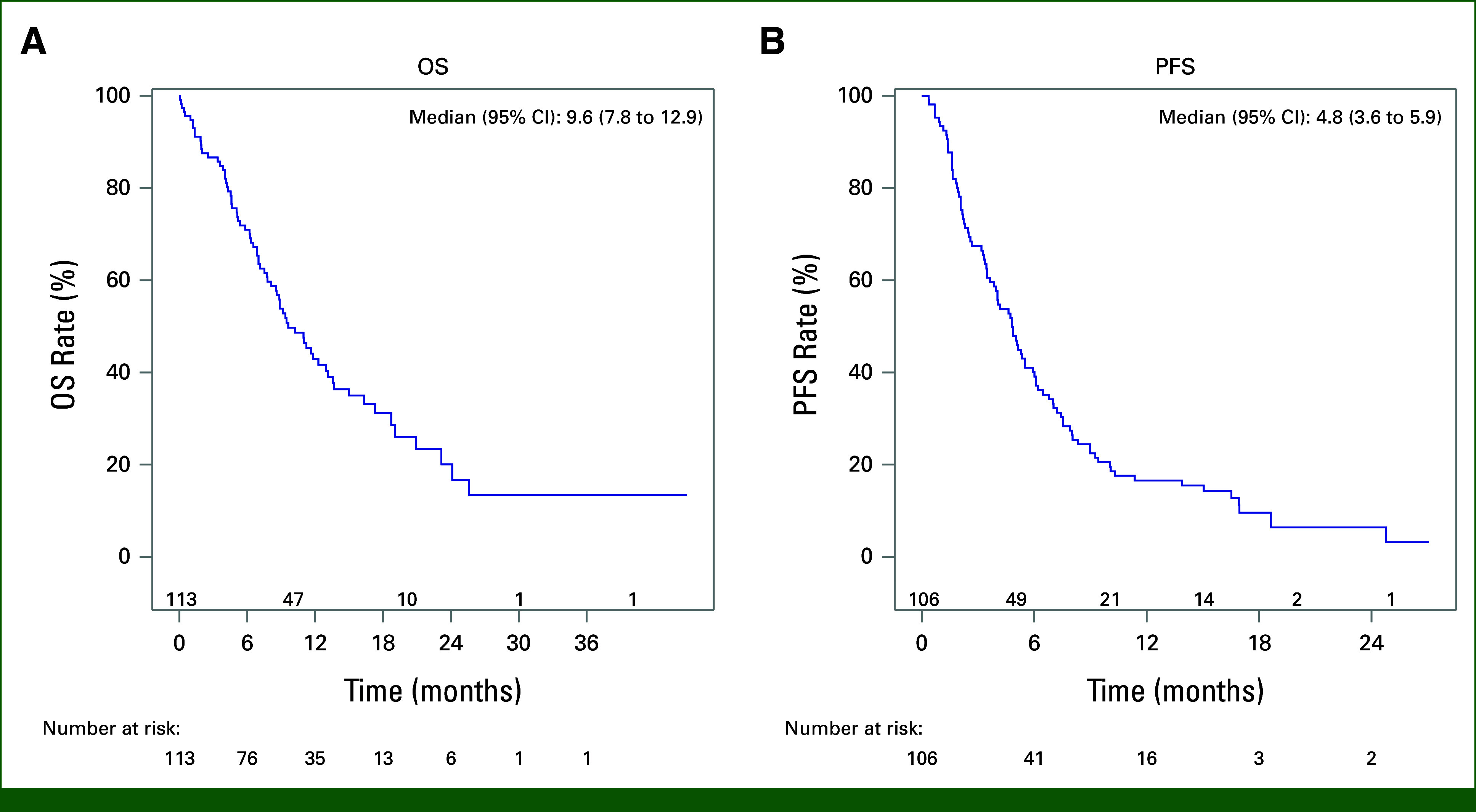
Survival outcomes in the overall patient population: (A) OS and (B) PFS. OS, overall survival; PFS, progression-free survival.

The median PFS was 4.8 months (95% CI, 3.6 to 5.9; Fig 2). RDI did not affect the response rates (median RDI in the PR/CR/SD *v* PD groups was 0.88 *v* 0.91; *P* = .3). The PFS of those who did not have primary refractory disease was 5.7 (3.3-8.3) months compared with 4.0 (2.2-5.1) months in patients with primary refractory disease (*P* = .02).

The median OS of the overall population was 9.6 months (95% CI, 7.8 to 12.9) with a median follow-up time of 16.1 months. The 1-year survival rate was 43% (95% CI, 33 to 52). The median DSS was consistent with the OS (9.6 months [95% CI, 7.8 to 12.9]; Fig 2). OS was higher in those who did not have primary refractory disease compared with those who had primary refractory disease (13.7 [8.9-25.6] *v* 8.1 [5.2-11.0] months; *P* = .02).

### Association Between Brain Metastasis and Survival Outcomes and AEs

There was no statistically significant difference in OS and PFS between patients with and without brain metastasis treated with SG**.** The occurrence of grade 3 and higher AEs was significantly higher in the brain metastasis group compared with the group without brain metastasis (*P* < .001). Treatment discontinuation due to AEs was significantly higher in the brain metastasis group (*P* = .015; Data Supplement, Table S2).

### Effectiveness of T-DXs After SG

**FIG 3.**
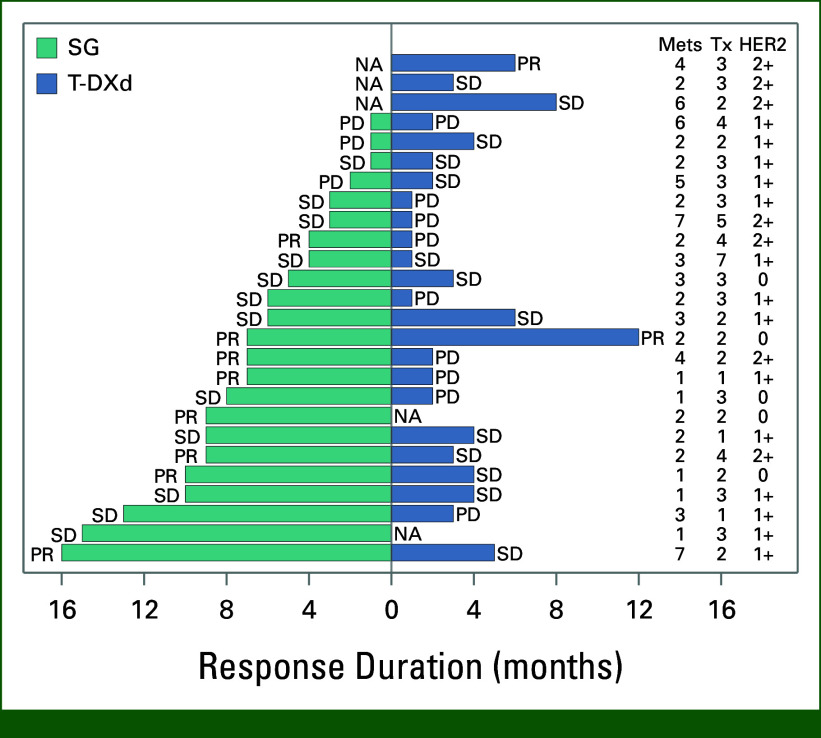
Efficacy of T-DXd after SG. HER2, human epidermal growth factor receptor 2; Mets, metastasis; NA, not applicable; PD, progression of disease; PR, partial response; SD, stable disease; SG, sacituzumab govitecan; T-DXd, trastuzumab deruxtecan; Tx, treatment.

Twenty-six patients (22.6%) with HER2-low breast cancer received T-DXd after SG therapy (among these, four patients had HER2 IHC zero at the time of biopsy of the metastatic disease site but were observed to have HER2-low disease during the initial diagnosis of breast cancer). In patients who had T-DXd after SG, the ORR was 34.8% (8/26; Fig 3) and CBR was 65% (15/26). Median PFS was 7 months (95% CI, 4.6 to 10.1), and the median follow-up time was 17.5 (95% CI, 15.6 to 17.6) months.

Among patients who had clinical benefit with SG and then received T-DXd, five patients (33.3%) experienced clinical benefit on T-DXd while 10 patients (67.7%) did not (*P* = .12). In this group, the PFS was 9.5 months (6.1-18.6) and the OS was not reached, with a lower limit of 13.6 months. Among those who did not experience clinical benefit with SG and subsequently received T-DXd (n = 8), none of the patients had clinical benefit. In this group, the PFS and OS were 3.4 months (1.3-4.7) and 8.9 months (4.0-9.6), respectively.

Three patients received T-DXd after discontinuing SG due to AEs such as grade 3 fatigue, anemia, and diarrhea. None of these side effects recurred after initiation of T-DXd.

### AEs

**FIG 4.**
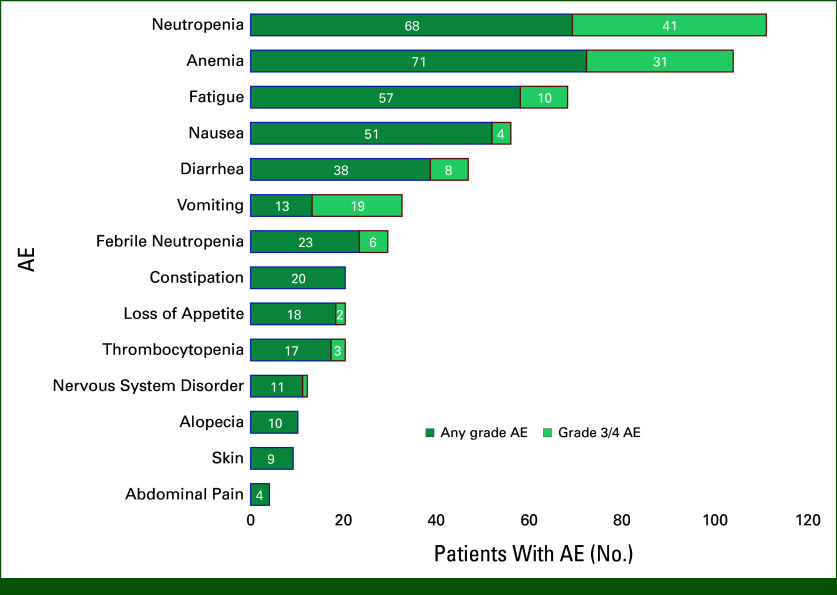
Rate of overall AEs and grade 3 AEs according to NCI-CTCAE v5.^17^ AE, adverse events; NCI-CTCAE, National Cancer Institute-Common Terminology Criteria for Adverse Events.

GCSF support was used by a total of 54 (47%) patients after starting SG, of whom 26 (22.6%) used primary GCSF support. Secondary GCSF prophylaxis was used by 28 (24.3%) patients, and the rest did not receive any GCSF prophylaxis. The rate of any grade AEs was 97.3% with grade 3 AEs seen in 57 (50.9%) patients (Fig 4). Overall, the most common AEs were anemia (61.7%), neutropenia (59.1%), fatigue (49.6%), nausea (44.3%), diarrhea (33.0%), and febrile neutropenia (20.0%). Dose reduction due to AEs was required in 58 patients (51.3%), while treatment discontinuation due to AEs occurred in 15 patients (13.2%). None of the patients in our study had UGT1A1 mutations.

**FIG 5.**
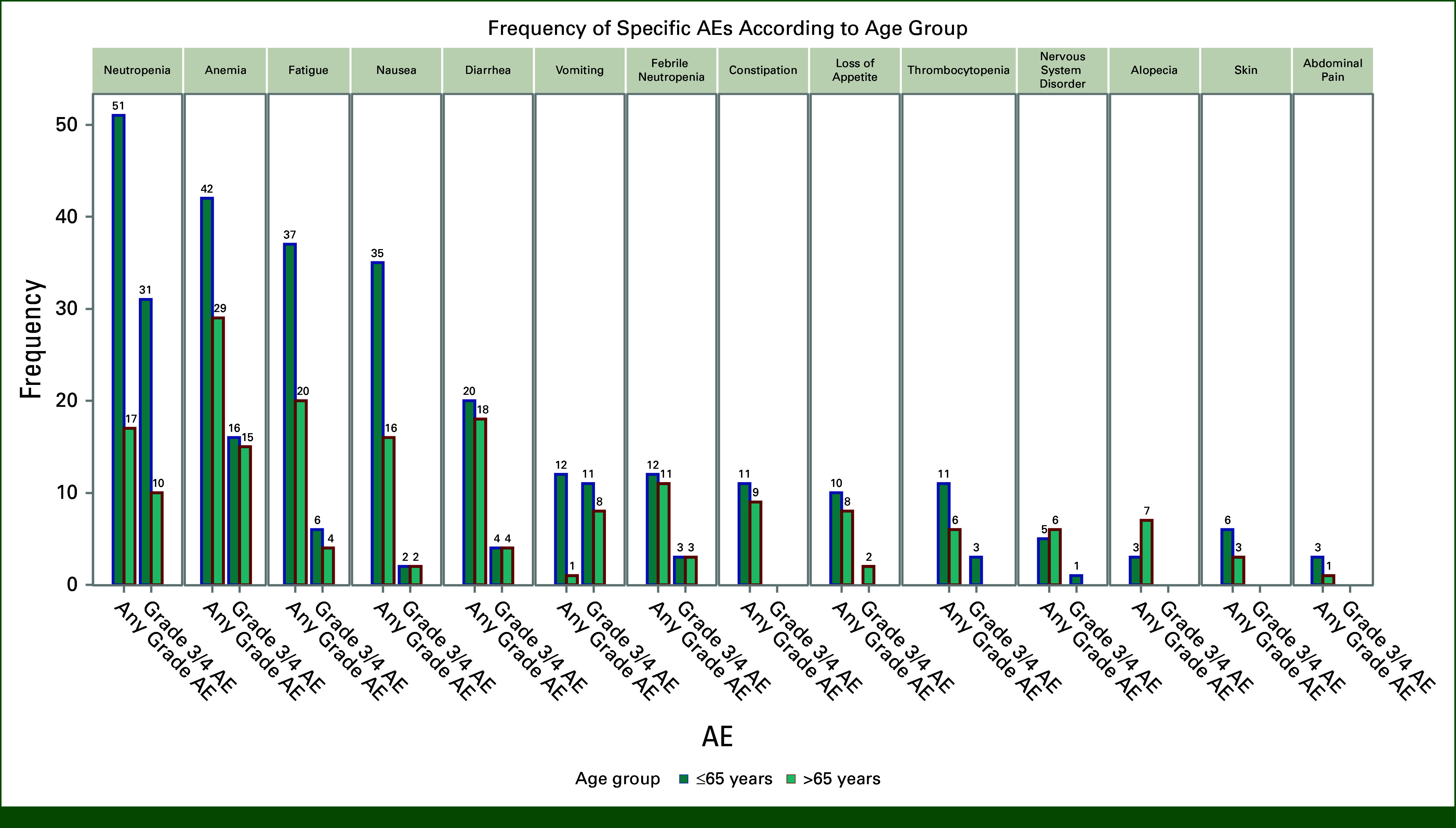
Rate of AEs and grade 3 AEs categorized by age dichotomy at 65 years. AE, adverse events.

There was no statistically significant difference in the incidence of grade 3 toxicity on the basis of the age of initiation of SG (≤65 years or >65 years). The rates of grade 3 toxicity were 40.8% and 25.6%, respectively, in these age groups (Fig 5).

## DISCUSSION

In our real-world report, we aimed to expand on the safety data of SG in a diverse patient population and compare real-world end points with those of the RCTs. In comparison with the ASCENT trial, our patients were slightly older, with a median age of 60 years at the time of SG initiation and 34% older than 65 years, whereas the trial group had a median age of 54 years with 20% of the trial population age 65 years and older.^7,18^ Our study population was more diverse, with 63.5% White and 27% Black patients compared with the ASCENT trial (White patients: 80%, Black patients: 12%).

The findings of this study indicate that most patients can be maintained on a high-intensity dose of SG. Notably, several patients had primary GCSF prophylaxis, although neither the trial nor FDA label recommend primary GCSF prophylaxis for neutropenia. This factor could have contributed to the preservation of dose intensity in this heavily pretreated group of patients. However, since the RDI was not observed to have an effect on response rates and nearly half of the patients required dose reductions, starting with a lower dose rather than the ASCENT dosing might have a greater impact on preserving the quality of life in heavily pretreated patients.

Neutropenia, anemia, vomiting, fatigue, and diarrhea were the most frequently reported clinically relevant grade 3 AEs in 51% of the patients. As a result, 51% of patients required dose reductions, while 13% required treatment discontinuation owing to the AEs. Interestingly, we observed that clinical benefit was still achieved even when the dose of SG was reduced. This finding aligns with the ASCENT study and further supports the recommendation of using an initial dosing of 10 mg/kg, followed by as-needed dose reductions for toxicities.^18,19^ UGT1A1 polymorphism (hinders SN-38 detoxification) is associated with higher rates of grade 3 AEs with SG.^18^ None of the patients in our study had this mutation.

The median number of previous anticancer regimens in the ASCENT study was three, including neoadjuvant/adjuvant therapy, and that in our study was two in the metastatic setting. Twenty-nine percent of patients in the trial received more than three lines of treatment.^7^ In our sample, 30% of patients had more than three lines of treatment for their metastatic disease excluding neoadjuvant/adjuvant therapy, indicating that we had similar proportion of heavily pretreated patients. Similar to the safety analysis from ASCENT, age at treatment initiation with SG was not associated with difference in grade 3 AE occurrence.^18^ However, our study population exhibited a higher rate of dose reductions compared with the ASCENT trial (51.3% *v* 22%). Additionally, the rate of treatment discontinuation was higher at 13% compared with 5% in the trial.^7^ Our findings align with other real-world studies, which have reported dose reduction rates ranging from approximately 39% to 54%.^20,21^

In terms of efficacy, we observed an ORR that is slightly lower than ASCENT (27% *v* 35%). The overall population had a median survival of 9.6 months (95% CI, 7.8 to 12.9) compared with ASCENT median OS of 12 months. The 1-year survival rate was also 43% (95% CI, 33 to 52), with a median PFS of 4.8 months (95% CI, 3.6 to 5.9). Our findings are consistent with other real-world data, which show a median real-world OS of 8.7-13 months, with a 1-year survival rate ranging from 40% to 52%.^20-22^

Early relapse and short disease-free interval in TNBC are associated with chemoimmunotherapy resistance and refractory disease, which confers a poor prognosis.^23-26^ Notably, 56% of the population in our study was diagnosed with primary refractory disease, which indicate aggressive disease behavior.^27,28^ We observed that patients with primary refractory disease were associated with poor PFS (4.0 *v* 5.7) and OS (8.1 *v* 13.7) compared with those who did not have primary refractory disease. Furthermore, we found that lines of previous therapy can influence duration of response, as the median response duration was shorter for patients who had received more than three lines of therapy compared with those who had received three or fewer lines (2 *v* 5 months; *P* = .012), Additionally, in line with the post hoc analysis from ASCENT, there was no statistically significant difference in CBR observed across the three HER2 status groups in our study.^29^

Regarding the sequencing of T-DXd after progression on SG, concerns exist because of the similar payload (both have topoisomerase I payload) and limited understanding of resistance mechanisms. None of the patients in our group had received T-DXd before SG. 26 patients (22.6%) received T-DXd after experiencing progression or toxicity on SG therapy. In the HER2-low subgroup who received T-DXd after SG, we observed a median PFS of 7 months, which is similar to the findings reported in DESTINY-Breast 04 (DB04) for the 40 patients with hormone receptor–negative breast cancer who received T-DXd (median PFS of 8.5 months).^14^ Among patients who had clinical benefit with SG and then received T-DXd, one third of the patients in our study had clinical benefit. Interestingly, among those who did not experience clinical benefit with SG and subsequently received T-DXd, none of the patients had clinical benefit. In a study by Abelman et al evaluating the response to sequential use of ADCs, it was observed that the median PFS for the first ADC was significantly longer compared with the second ADC. Also, the cross-resistance appeared to improve when a different antibody target was used.^30^ Although the small sample size of our study and retrospective nature are notable limitations, these findings do provide some reassurance that T-DXd could be a viable option after SG, especially for those who had clinical benefit with SG. Large-scale studies with prospective designs are needed to further validate these results.

Numerous ongoing studies are exploring novel ADCs and investigating their sequencing to better comprehend both efficacy and toxicity when administered consecutively. Among these, the phase III trial TROPION-Breast 02 is assessing the efficacy of the new ADC datopotamab deruxtecan (anti-TROP2 IgG1 monoclonal antibody linked to a topoisomerase I inhibitor payload) in previously untreated patients with mTNBC ineligible for immune checkpoint inhibitors.^31^ Similarly, the TRADE-DXd trial is planning to investigate the optimal sequencing of ADCs, specifically by comparing datopotamab deruxtecan and T-DXd in patients with locally advanced or metastatic HER2-low breast cancer.^32^ The findings from these trials will contribute to the availability of newer ADCs for breast cancer treatment, providing stronger evidence regarding their sequencing.

Our study has some limitations. First, the sample size in our study was relatively small, which restricts the generalizability of our findings to larger patient populations. Moreover, our study used retrospective data that usually exhibit variations in data quality and completeness with varying intervals of imaging per physician discretion. It is important to acknowledge that selection bias as an inherent limitation of real-world studies. Despite these limitations, our study contributes to the expanding body of knowledge on SG and provides reassurance to oncologists regarding its efficacy, alleviating concerns that it may not achieve the same level of effectiveness observed in the ASCENT trial.

In conclusion, the real-world effectiveness of SG aligns with the trial results, albeit with a higher incidence of toxicity and dose modifications. Given that RDI of SG did not affect the response rates and as dose reductions were common, starting treatment with a lower dose may be considered for heavily pretreated patients. The findings suggest that SG can be effective in patients with primary refractory disease, offering a valuable treatment option for this challenging population. The sequential use of T-DXd after SG exhibits promise in the treatment of HER2-low subset of mTNBC. Further research is needed to explore optimal sequencing strategies with other agents including T-DXd to guide clinical decision making.

## Data Availability

The data sets used and/or analyzed during the current study are available from the corresponding author on reasonable request. Analysis code is available from the corresponding author upon reasonable request.
